# The Verification of the Usefulness of Electronic Nose Based on Ultra-Fast Gas Chromatography and Four Different Chemometric Methods for Rapid Analysis of Spirit Beverages

**DOI:** 10.1155/2016/8763436

**Published:** 2016-06-29

**Authors:** Paulina Wiśniewska, Magdalena Śliwińska, Jacek Namieśnik, Waldemar Wardencki, Tomasz Dymerski

**Affiliations:** Department of Analytical Chemistry, Faculty of Chemistry, Gdańsk University of Technology, 11/12 Gabriela Narutowicza Street, 80-233 Gdańsk, Poland

## Abstract

Spirit beverages are a diverse group of foodstuffs. They are very often counterfeited which cause the appearance of low quality products or wrongly labelled products on the market. It is important to find a proper quality control and botanical origin method enabling the same time preliminary check of the composition of investigated samples, which was the main goal of this work. For this purpose, the usefulness of electronic nose based on ultra-fast gas chromatography (fast GC e-nose) was verified. A set of 24 samples of raw spirits, 33 samples of vodkas, and 8 samples of whisky were analysed by fast GC e-nose. Four data analysis methods were used. The PCA was applied for the visualization of dataset, observation of the variation inside groups of samples, and selection of variables for the other three statistical methods. The SQC method was utilized to compare the quality of the samples. Both the DFA and SIMCA data analysis methods were used for discrimination of vodka, whisky, and spirits samples. The fast GC e-nose combined with four statistical methods can be used for rapid discrimination of raw spirits, vodkas, and whisky and in the same for preliminary determination of the composition of investigated samples.

## 1. Introduction

Electronic noses are more and more often taken into consideration as a tool used for the quality and authenticity assessment of selected products due to their numerous advantages and the principle of operation. The electronic nose is an analytical instrument used for rapid detection and differentiation between types of gaseous samples. Till now electronic nose was used for environmental monitoring (detection of air pollution, tracking of pollution pathways, and efficiency assessment of wastewater and waste gas treatment), medical sciences (identification of selected diseases, including tumors, based on odorants excreted by the infected cells and organs), the perfume industry (authentication of perfumes), the pharmaceutical industry (production control of medicines), forensic operations, and the food-processing industry [1]. This instrument mimics the principles of operation of human smell. Most of the electronic noses are based on set of sensors. Chemical sensors under the influence of the gas mixture allow for generating a characteristic odour profile, which constitutes the so-called “fingerprint” [2]. Sensors such as piezoelectric, electrochemical, and biosensors are usually used. Each chemical sensor has some limitations. For example, metal oxide semiconductor (MOS) sensors require a high working temperature, and conductive polymer (CP) sensors are susceptible to humidity. These problems can be solved by use of e-noses based on the ultra-fast gas chromatography or mass spectrometry. Example of e-nose based on the ultra-fast gas chromatography technology is Heracles II. This type of electronic nose allows for combining the features of fast gas chromatography (fast GC) with advantages of a sensor-based electronic nose. In this electronic nose system, hundreds of variables can be used to achieve reliable results. As a result of one analysis, it is possible to obtain information about the composition of investigated samples and their volatile fraction profile. In this way, complete information is obtained about the similarity of a given sample to the reference sample [3]. An electronic nose based on ultra-fast gas chromatography can be used to assess quality and authenticity and to compare samples.

Spirit beverages are a diverse group of foodstuffs, which differ in terms of their taste, odour, and appearance. Spirit beverages which are produced from grain distillates, namely vodka and whisky, belong to the most popular spirit beverages. For production of vodka and whisky is used the raw spirit which according to Regulation number 110/2008 of the European Parliament and of the Council (EC) of 15 January 2008 is obtained by the distillation, after alcoholic fermentation, which does not have the characteristics of ethyl alcohol or a spirit drink but still retains the odour and flavour from the specific agricultural materials. The composition of agricultural distillates is influenced by the material, from which it is produced, and fermentation conditions [4–6]. Raw spirits may contain a lot of harmful substances, which during the production process can pass to the vodka and whiskey. Moreover, agricultural distillates can be falsified due to their botanical origin, for example, by mixing rye distillates with corn distillates [7].

Vodka is a very popular alcoholic beverage in Eastern European countries. Ethyl alcohol of agricultural origin is one of the materials used for producing vodka. Ethanol obtained during the fermentation of various agricultural materials is distilled to selectively reduce the organoleptic properties of materials [6]. By filtering alcohol several times through charcoal and by diluting it with water, an alcoholic beverage with a mild flavour is obtained [8, 9]. Whisky, just like most of the vodkas, is a grain spirit beverage produced by the distillation of a mash made from malted cereals or cereals saccharified as a result of diastasis of malt contained in them. The flavour of whisky is closely connected with the distillation process and maturation of the intermediate product in oak barrels for at least three years, which is different from vodka. Only water and pure caramel can be added to the distillate for dilution and coloring, in contrast to vodkas, to which aromas can be added; however, in this case the name of aroma should be marked on the label, for example, if it is peach aroma on the label, it should be written as “peach vodka” [10]. The composition of vodkas and whisky is usually analysed using one-dimensional gas chromatography [11–17] or two-dimensional gas chromatography [18]. If less volatile ingredients are determined, such as coumarin, it is also possible to use the HPLC technique [19]. Quality and authenticity assessments are also conducted for these matrices using infrared spectroscopy [20–22], optical spectroscopy [23], and sensory analysis [24–26] and the use of the electronic nose based on sensors [27]. None of these methods allows the simultaneous investigation of the matrix and quick differentiation of samples. Due to their diversity and popularity, spirit beverages are very often counterfeited. Lower quality products are defined as those of better quality. Raw materials on the labelling differ from those used in reality. Because of this it is important to find a method that would enable the distinction of samples because of the quality and origin and in the same time a preliminary check of the composition of the samples. Such method should be quick which would allow its introduction to the production lines.

There is a need to find equipment that quickly delivers information about composition and authenticity of spirit beverages. These requirements are fulfilled by Heracles II electronic nose based on ultra-fast gas chromatography. Two of the most popular spirit beverages, namely, vodka and whiskey, and raw spirits used for their production were selected to check the suitability of the electronic nose for quality analysis of spirit drinks. These alcoholic beverages differ significantly from each other by methods of production but they are made from similar raw materials; due to this fact it will be possible to check whether one method is good for a variety of spirits made from grains. 

## 2. Materials and Methods

### 2.1. Materials

A set of 24 samples of raw spirits made from different grains obtained from Destylarnia Sobieski S.A. (Poland, Pomeranian Province) were used for the research. Samples of commercially available vodkas and whisky were obtained from local stores in Gdańsk, Poland. A set of 33 samples of vodkas produced from different mixture of different type of rye were selected. Eight brands of whisky were selected from those available on the market, out of which 5 ones were produced from a mixture of barley, rye, and wheat distillates and three whiskies were produced with the addition of maize distillate.

### 2.2. Methods

#### 2.2.1. Sample Preparation

All samples were prepared using the same analytical procedure. To the vials of 20 mL 6.25 mL of deionised water and 1.75 mL of the alcoholic sample (vodka, whisky, or raw spirit) were added. Next, vials were incubated for 20 minutes at 40°C. Samples were dispensed using a syringe kept at 100°C.

#### 2.2.2. Instrumentation

The Heracles II electronic nose based on ultra-fast gas chromatography by Alpha MOS (Toulouse, France) was used for the research. It is equipped with a sorption trap, a dispenser allowing the introduction of a gas or liquid sample, two Flame Ionisation Detectors (FID), dedicated AlphaSoft V12 software with implemented modules for chromatographic, chemometric, and sensory analysis of characteristics of detected chemical compounds, the Arochembase V4 library, an HS100 autosampler, and a set of independent chromatographic columns with different polarity (nonpolar MXT-5 and medium polar MXT-1701, length 10 m each). Parameters of operation of the electronic nose used for the analysis of spirit beverages are presented in Table 1.

## 3. Results and Discussion

**Figure 1:**
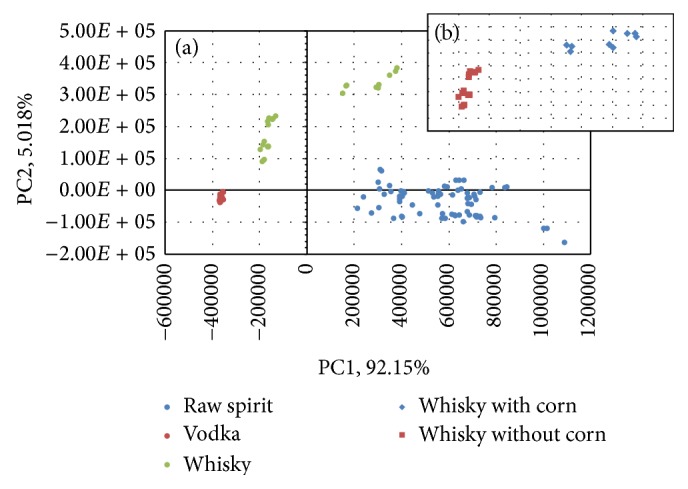
(a) PCA results for vodkas, whiskies, and raw spirits; (b) PCA results for vodkas, whiskies made from corn, whisky made without corn, and raw spirits.

**Figure 2:**
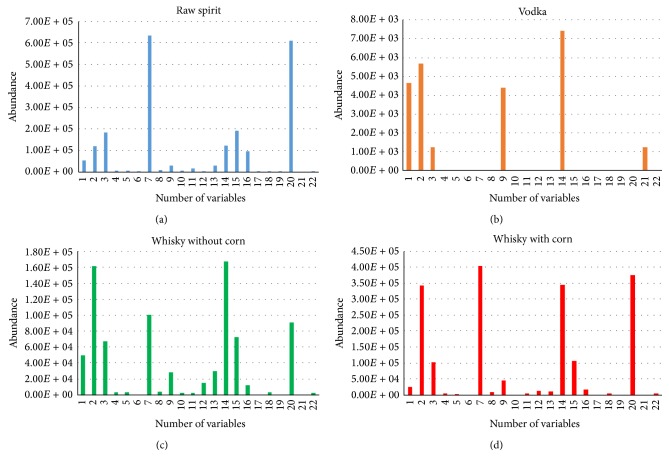
Mean bar graphs of selected peak areas used as raw data in chemometrics representing key chemical compounds, which are important for discrimination of (a) raw spirit, (b) vodka, (c) whisky without corn, and (d) whisky with corn.

Following chemometric analysis PCA (Principal Component Analysis), DFA (Discriminant Function Analysis), SIMCA classification (SIMCA (Soft Independent Modeling of Class Analogies)), and SQC (Statistical Quality Control) were used for data analysis. For all analyses, sensors (area of the peaks) with the highest discrimination power were used. At the first stage, PCA was used. Principal Component Analysis is one of the most popular chemometric methods used for modelling, compression, and visualization of multidimensional data [28, 29]. Because of that, this method was checked for visualization of the obtained data. All beverages listed in point 2.1 were used for data analysis (Figure 1). PCA allowed for distinguishing between all the three groups of samples. However, this analysis is not typically used to distinguish between groups; it allows spotting the differences between the analysed samples. For example, in Figure 1(a), how diversified the studied groups are can be seen. Groups were created due to the type of spirit drinks (raw spirits, vodka, and whisky). On the basis of the distance between the samples, it can be concluded that the group of whisky comprises two substantially different groups of samples. In the case of raw spirit samples, the relatively low precision of results is caused by the fact that all samples were made from unknown mixture of grains. All vodkas were made from the same type of grain, namely, rye. In the case of whisky, the information about botanical origin of samples was available. Three of them were made not only from barley, wheat, and rye, but also from corn. Because of that, two separate groups of whisky of different botanical origin were created (Figure 1(b)). The variables chosen for PCA, namely, the most discriminant peak areas of specific compounds, were treated as an input dataset for statistical analysis. This dataset was presented in Figure 2 and the names of selected substances were listed in Table 2. Due to satisfactory discrimination of sample groups after application of PCA method, chosen variables were also used as input data in other utilized chemometric methods.

**Figure 3:**
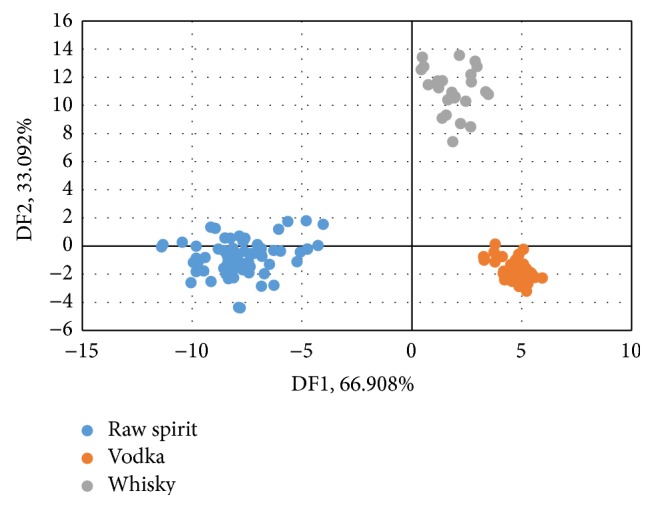
DFA of vodkas, whiskies, and raw spirits.

**Figure 4:**
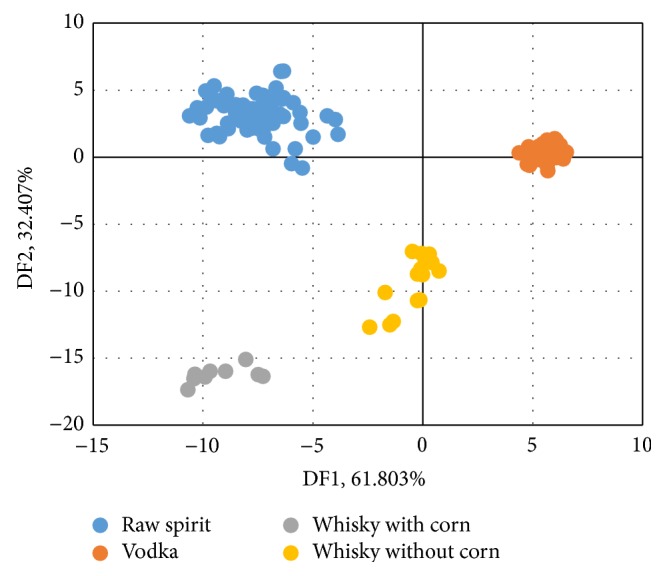
DFA results for vodkas, whiskies made from corn, whisky made without corn, and raw spirits.

Discriminant Function Analysis is one of the most commonly methods used to decide which variables allow correct classification of a set of objects [30, 31]. DFA was used for discrimination of the data obtained from e-nose analysis of spirit beverages. In Figure 3 the results of DFA analysis for groups of vodkas, whiskies, and raw spirits are shown, and in Figure 4 the results of DFA for vodkas, whiskies made from corn, whisky made without corn, and raw spirits are shown. Both DFA allowed for distinguishing between all groups of samples. As opposed to PCA, DFA is used to distinguish groups not to make the actual data visualization. In Figure 3 it can be seen that points belonging to group of whiskies are definitely more concise than in the case of PCA. The groups of samples were not divided. Using DFA data analysis method the differentiation between groups of samples is more unambiguous. On the other hand differences between individial samples are disappearing when a grup of whiskey samples is defined as a one coherent group. When two groups of whiskies are created (groups which were showed on the PCA graph (Figure 1(b)) it can be seen that the groups are distinguished very well (Figure 4). In the case of DFA, spirit beverages can be distinguishing due to the general type (raw spirits, vodka, and whisky) and due to the botanical origin if the information about their origin is known as in the case of whisky produced with and without corn. In the case of the PCA the differences between the samples within the selected groups cannot be neglected.

**Figure 5:**
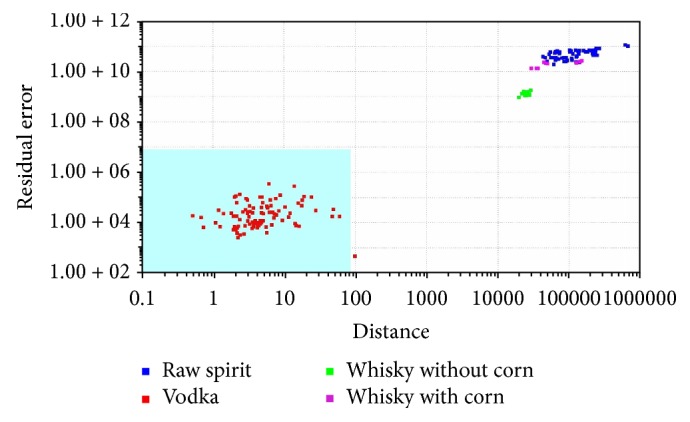
SIMCA analysis for vodkas, whiskies, and raw spirits: vodka, reference group.

**Figure 6:**
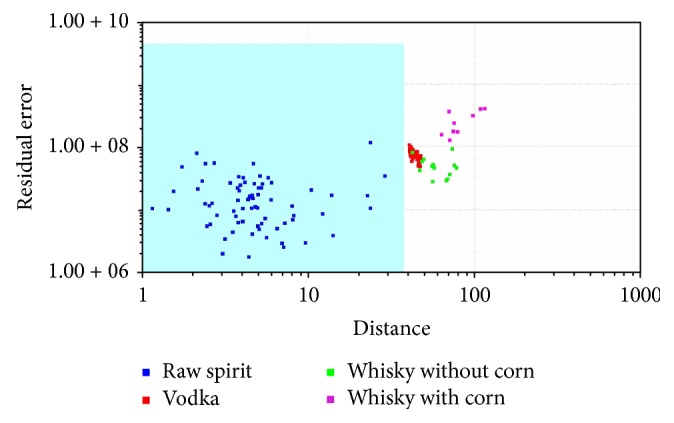
SIMCA analysis for vodkas, whiskies, and raw spirits: raw spirit, reference group.

**Figure 7:**
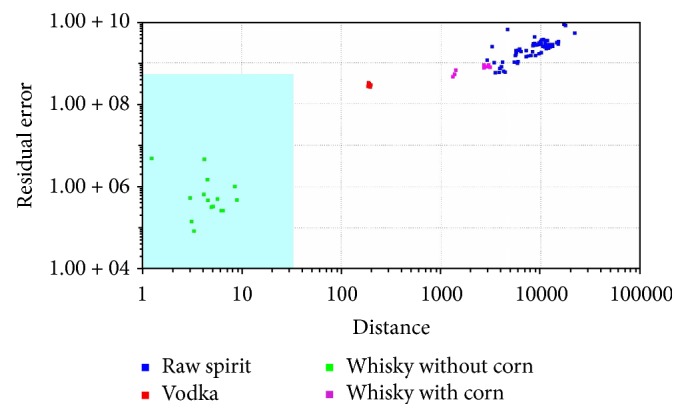
SIMCA analysis for vodkas, whiskies, and raw spirits: whisky without corn, reference group.

**Figure 8:**
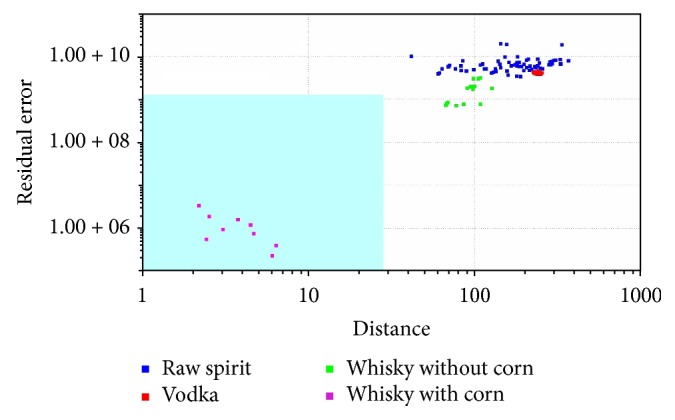
SIMCA analysis for vodkas, whiskies, and raw spirits: whisky with corn, reference group.

In SIMCA classification, a separate model is created for each class based on the principal component method. Next, the so-called “confidence envelope” (a certain volume) is created around the model. It should include with defined probability all elements belonging to a given class [1, 32]. SIMCA analysis is method typically used for classification of the samples. It was used for classification samples into four groups. SIMCA analysis was made for each reference group: vodka (Figure 5), raw spirit (Figure 6), whisky without corn (Figure 7), and whisky with corn (Figure 8). In all cases with one exception points belonging to the reference group were located in the blue area in the confidence envelope. In the case of exception one sample was not classified properly to the reference group in case of vodka classification. This analysis is similar to DFA and it is used to distinguish the groups. In contrast to DFA, SIMCA graph is created for each group separately. It is therefore more time-consuming than DFA. On the other hand, discrimination between different groups is more visible. Due to the envelope of confidence the boundaries of analysed groups are determined. Therefore, the identification of unknown samples is unambiguous.

**Figure 9:**
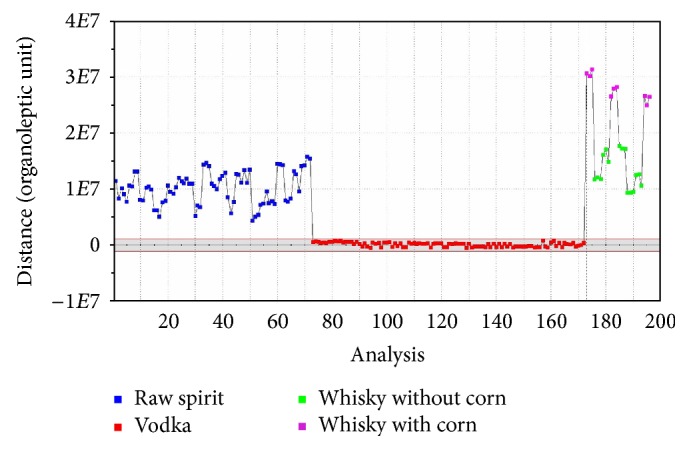
SQC analysis for unflavoured vodkas, raw spirits, and whiskies: vodka, reference group.

**Figure 10:**
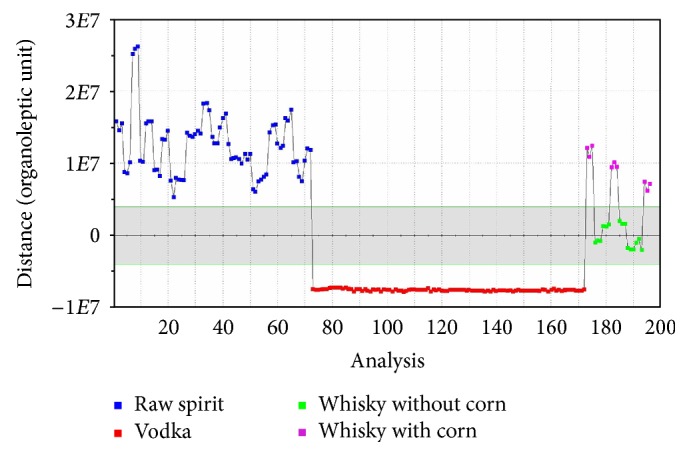
SQC analysis for unflavoured vodkas, raw spirits, and whiskies: whisky without corn, reference group.

The SQC analysis is used for process quality control. Samples, which belong to one group, should be situated in the area designated using parameters on the *y*-axis of the graph [33, 34]. It can be used for quality analysis of samples on production lines. SQC analysis was performed for four groups of samples, namely, for vodkas, whiskies without corn, whiskies with corn, and raw spirits to show capabilities of this method. SQC analysis allows seeing the differences between individual samples and excluding those that differ from others. It is the proper method for assessing the quality of products, because it is rapid and easy to use and the discrete differences in the studied group of samples can be determined. This method allows observing which sample is statistically different from the other ones in a given batch. It enables the possibility of exclusion of a sample during the final control of ready products. In Figure 9 it can be seen that the differences between the points assigned to a group of vodkas are small. In contrast, the differences between the points belonging to the group of whiskies without corn in Figure 10 are more noticeable. It can be noticed that the area in which points belong to the reference group should be located depending on the deviation between the samples in certain group. The greater the differences between the samples in the group, the greater the tolerance range for the whole group.

**Figure 11:**
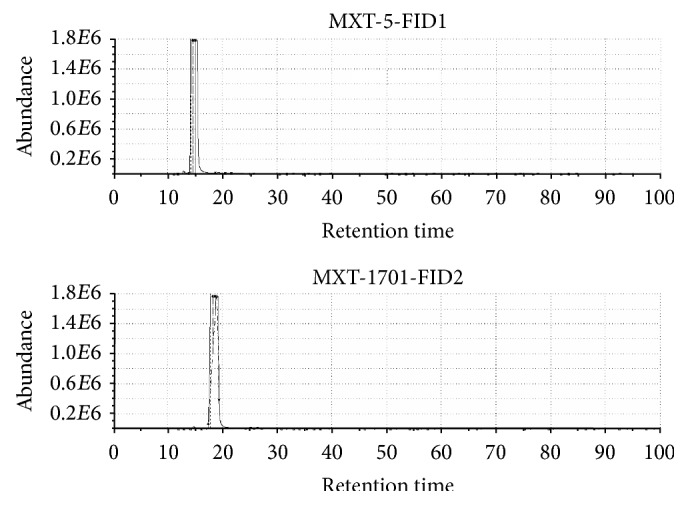
An example of a vodka sample presented in a chromatogram.

**Figure 12:**
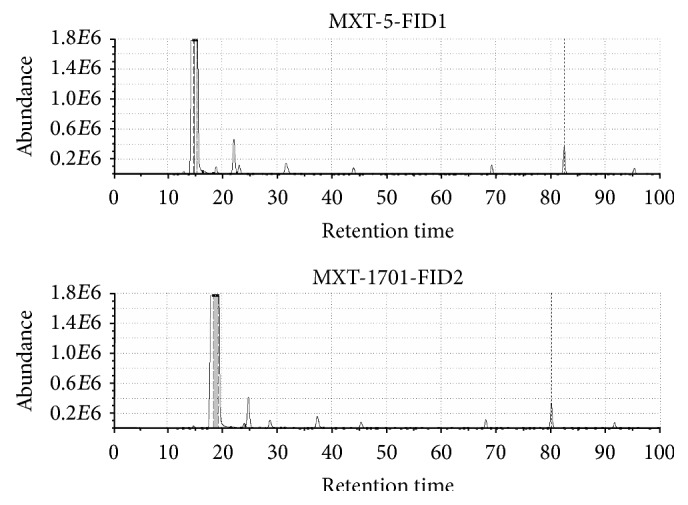
An example of a whisky sample presented in a chromatogram.

Due to the presence of two chromatographic columns of different polarity, it is possible to obtain two different chromatograms and identify individual compounds included in the samples. This is the difference between electronic nose based on sensors and electronic nose based on fast GC. Figures 11 and 12 present chromatograms obtained from the analysis of vodka and whisky. It can be noticed that they differ considerably from each other. As compared to vodka, the odour profile of whiskey is much richer. Nearly twice as many compounds were detected for whisky compared to vodka. Some of chemical compounds detected in vodka, whisky, and raw spirits samples are presented in Tables 3, 4, and 5, respectively. Criterion for the selection of compounds was the similarity parameter set at 90%. Compounds present in the sample were identified on the basis of comparison of calculated linear temperature-programmed retention indices (LTPRI) for compounds from both columns with indices provided in the literature. Apart from LTPRI, the tables also include approximate retention times for the detected chemical compounds and odour descriptions contained in literature sources, which can be found in Arochembase V4 library. Compounds present in the volatile fraction of vodka can be characterized by fewer descriptors than those found in whisky and raw spirits. Mostly, the aroma of compounds identified in vodka matrix was defined as fruity or green, but some of them were also described as sulphurous, winey, alcoholic, bread, straw, corn, or alkane one. In the case of whisky, compounds are mostly characterized as fruity, green, and woody. Other compounds were described as spicy, winey, earthy, sweet, smoky, alcoholic, or pine. Regarding the aroma of raw spirits most of the constituents of this matrix were determined as fruity, fusel, and anise one. Some of the compounds were described as a green, woody, minty, floral, sweet, and solvent one. As it can be noticed the decryption of compounds present in the whisky samples was more connected with aroma of barrels, forest, and sweet fruits than in the case of vodka and raw spirit samples.

## 4. Conclusion

In this paper the usefulness of the electronic nose using ultra-fast gas chromatography for the qualitative analysis of selected spirit beverages was checked. Four chemometric methods were used to interpret obtained results. PCA and SQC analysis were used to check deviations of individual samples from groups to which they belong. PCA enables the visualization of results. As a result, it was possible to quickly determine that the group of whiskies includes two different groups of samples. Through an SQC analysis, it is possible to check the quality of the samples. Samples of vodka had a similar composition and method of production, due to this fact they are located in one line in Figures 9 and 10. The differences in composition between whisky samples are more significant due to their different method of production and different raw materials used, which was presented in Figures 9 and 10. DFA and SIMCA analysis, on the other hand, were used to group vodka, whisky, and spirits samples and to compare them. Furthermore, in contrast to traditional electronic nose based on the sensors, the electronic nose based on a fast gas chromatography allows determining the compounds included in the sample. This is possible thanks to the presence of two chromatographic columns of different polarity and well-equipped database that allows comparison of retention indexes and retention times. Additionally, each compound can be attributed to the smell which has been previously described in the literature. Summing up, the use of electronic nose based on ultra-fast gas chromatography and four applied statistical methods can be used to distinguish groups of spirit beverages. It also allows finding the differences between individual samples in one group and to determine the composition of the investigated samples. By using Arochembase V4 library it was possible to make an assignment of the odours described in the literature to the individual components present in the samples.

## Figures and Tables

**Table 1:** Parameters of electronic nose operation.

Parameter	Conditions
Dispenser operation conditions	
Dispensing volume	2.5 mL
Dispensing time	15 s
Dispenser temperature	200°C
Volumetric intensity of carrier gas flow	30 mL/min

Parameters of sorption trap operation	
Trap temperature	40°C
Retention time	20 s

Conditions for chromatographic analysis	
Temperature programme	40°C (2 s) - 3°C/s - 270°C (18 s)
Carrier gas	Hydrogen
Dispenser temperature	270°C

Detector operation conditions	
Detector temperature	270°C

**Table 2:** Key chemical compounds (variables), which are important for sample discrimination.

Number of variables from – 1 or 2 column	Name of the compound	Type of sample (raw spirit, vodka, whisky without corn, and whisky with corn)
1—1	tert-Butylmethylether	All
2—1	Ethyl acetate	All
3—1	2-Methyl-1-butanol	All
4—1	2-Methyl-1-propanol	Raw spirit, whisky without corn, and whisky with corn
5—1	n-Butanol	Raw spirit, whisky without corn, and whisky with corn
6—1	2,3-Pentanedione	Raw spirit
7—1	Methyl butanoate	Raw spirit, whisky without corn, and whisky with corn
8—1	Pentanol	Raw spirit, whisky without corn, and whisky with corn
9—1	1-Hexanol	All
10—1	(Z)-4-Heptenal	Raw spirit and whisky without corn
11—2	Diethyl ether	Raw spirit, whisky without corn, and whisky with corn
12—2	2-Methylfuran	Raw spirit, whisky without corn, and whisky with corn
13—2	2-Methyl-1-propanol	Raw spirit, whisky without corn, and whisky with corn
14—2	Ethyl acetate	All
15—2	Methyl butanoate	Raw spirit, whisky without corn, and whisky with corn
16—2	Propyl acetate	Raw spirit, whisky without corn, and whisky with corn
17—2	1-Hydroxy-2-propanone	Raw spirit
18—2	1-Hexen-3-ol	Raw spirit, whisky without corn, and whisky with corn
19—2	2-Methylthiophene	Raw spirit
20—2	Pentanol	Raw spirit, whisky without corn, and whisky with corn
21—2	3-Methylbut-2-en-1-ol	Vodka
22—2	(Z)-3-Hexen-1-ol	Raw spirit, whisky without corn, and whisky with corn

**Table 3:** Compounds detected in vodka samples.

Name	RT1/LTPRI1	RT1/LTPRI2	LTPRI1 LIT	LTPRI2 LIT	Descriptor
(E)-2-Heptenal	50.41/950	53.00/1049	960	1062	Sulfurous, earthy, grossy, and almond
(E)-2-Octene	38.55/813	34.72/816	815	819	—
(Z)-2-Nonenal	64.94/1134	66.97/1251	1148	1254	Cucumber, *geranium* and green
**1-Hexanol**	42.59/875	45.73/991	870	980	Dry, floral, grossy, green, woody, and fruity
1-Octen-3-one	52.82/979	54.96/1075	979	1066	Herbaceous
2-Methyl-1-butanol	32.07/739	37.35/848	739	852	Winey, buttery, and malty
3-Methylbut-2-en-1-ol	35.22/775	38.53/863	778	863	Fruity and green
3-Methylfuran	22.07/614	37.44/849	614	856	—
**Ethanol**	14.91/426	18.55/575	437	564	Alcoholic
**Ethyl acetate**	21.00/600	24.68/682	609	673	Acidic, etheral, fruity, and orange
Ethyl butyrate	37.31/799	38.42/861	799	864	Fruity and acetone
Furfural	38.49/812	46.80/967	827	972	Almond and bread
Hexyl butyrate	69.28/1193	68.24/1271	1191	1257	Apple, fruity, and green
Limonene	58.06/1044	54.82/1074	1033	1061	Citrus, fruity, and minty
**Methanol**	14.63/418	16.67/516	419	507	—
Methylnonanedione	73.97/1261	75.92/1396	1253	1397	Fruity and straw
**Propenal**	15.24/435	18.11/561	450	566	—
Pyrazine	31.11/728	35.94/831	738	822	Corn and nutty
Tert-butylmethylether	19.31/551	19.24/596	546	600	—
Tetradecane	82.64/1393	75.83/1395	1400	1400	Alkane, sweet, and mildly herbaceous
Undecane	62.56/1101	56.14/1091	1100	1100	Alkane and fusel

RT1: retention time in column 1 (MXT-5);

LTPRI1: linear temperature-programmed retention index for compounds from column 1;

RT2: retention time in column 2 (MXT-1701);

LTPRI2: linear temperature-programmed retention index for compounds from column 2;

LTPRI1 LIT: linear temperature-programmed retention index for compounds from column 1 from literature;

LTPRI2 LIT: linear temperature-programmed retention index for compounds from column 2 from literature.

The positive identification of selected compounds was highlighted in bold font.

**Table 4:** Compounds detected in whiskey samples.

Name	RT/LTPRI1	RT/LTPRI2	LTPRI1 LIT	LTPRI2 LIT	Descriptor
(E)-2-Decanal	73.93/1260	74.36/1370	1262	1371	Green, orange, and tallowy
(E)-2-Heptenal	51.87/967	54.44/1068	960	1062	Almond, earthy, grossy, and sulfurous
(Z)-3-Hexen-1-ol	41.95/851	45.39/949	852	960	Mossy, green, and fresh
(Z)-3-Hexen-1-ol, butanoate	62.83/1173	67.05/1252	1187	1256	Banana, green, and winey
(Z)-3-Hexenyl isobutyrate	64.91/1133	64.39/1211	1144	1208	Apple, etheral, sweet, and winey
(Z)-4-Heptenal	46.43/903	48.33/986	900	988	Creamy, sweet, and boiled potato
(Z)-Whisky lactone	76.94/1305	84.64/1550	1316	1548	Coconut
**1-Hexanol**	44.01/875	48.67/991	870	980	Dry, floral, grossy, green, woody, and fruity
1-Hexen-3-ol	34.00/772	38.82/865	775	850	Green
2, 4, 5-Trimethylthiazole	54.21/995	54.50/1069	996	1073	Earthy, hazelnut, moldy, and chocolate
2-Methyl-1-butanol	31.68/735	37.41/849	739	852	Fruity and malty
**2-Methyl-1-propanol**	23.13/628	28.76/740	628	735	Alcoholic, bitter, and winey
**2-Methylfuran**	21.06/601	22.64/652	602	639	Burnt, solvent, metallic, and musty
2-Methylphenol	57.98/1043	69.35/1288	1054	1283	Phenolic
3, 5-Octadien-2-one	61.96/1093	64.00/1205	7092	1203	Fruity and mushroom
3-Heptanone	45.39/891	46.91/968	888	969	Cinnamon, green, spicy, and sweet
4-Ethylguaiacol	74.66/1271	77.35/1421	1282	1430	Floral, spicy, and phenolic
6-Decenal	70.20/1206	70.60/1308	1203	1294	Green
**Benzaldehyde**	50.37/950	55.86/1088	959	1086	Almond, woody, and burnt sugar
**Beta-ionone**	88.65/1487	88.71/1623	1490	1627	Floral, woody, and raspberry
Beta-phellandrene	56.63/1026	52.75/1045	1030	1059	Fruity, minty, and herbaceous
**Beta-pinene**	52.71/977	48.79/992	979	994	Green, pine, sweet, and resin
Butanoic acid	38.50/812	46.84/967	816	970	Rancid, sweaty, and butter
Carvone	73.14/1249	75.94/1397	1253	1386	Minty and peppermint
Diethyl ether	16.70/476	16.90/524	491	532	—
**Ethanol**	15.14/432	18.28/566	437	564	Alcoholic
**Ethyl acetate**	22.16/615	24.05/675	609	673	Acidic, etheral, fruity, and orange
Ethyl butyrate	37.34/799	38.92/867	799	864	Banana, fruity, sweet, and acetonic
Furfural	40.86/839	47.61/977	827	972	Almond, bread, and sweet
Geosmin	85.91/1444	83.15/1523	1430	1513	Beet and earthy
Hexyl acetate	55.27/1009	55.80/1087	1011	1082	Citrus, fruity, green, and spicy
Hexyl butyrate	69.27/1193	68.18/1270	1191	1257	Apple, fruity, and green
Indole	76.04/1291	84.64/1550	1295	1549	Burnt, earthy, floral, and jasmine
Limonene	56.63/1026	53.13/1051	1033	1061	Citrus, fruity, minty, and peely
Methyl butanoate	29.59/711	31.73/778	717	784	Ester, etheral, green, and sweet
n-Butanol	26.00/655	31.69/774	651	778	Fermented and fruity
p-Cresol	60.18/1071	71.60/1325	1072	1312	Phenolic and smoky
Pentanol	33.68/757	38.87/867	767	879	Anise, fruity, and green
Propyl acetate	28.76/701	31.38/774	708	775	Fruity, ketonic, sweet, and solvent
Terpinolene	61.36/1086	58.04/1118	1088	1112	Fruity, pine, herbaceous, and sweet
tert-Butylmethylether	20.35/551	20.28/596	546	600	—

RT1: retention time in column 1 (MXT-5);

LTPRI1: linear temperature-programmed retention index for compounds from column 1;

RT2: retention time in column 2 (MXT-1701);

LTPRI2: linear temperature-programmed retention index for compounds from column 2;

LTPRI1 LIT: linear temperature-programmed retention index for compounds from column 1 from literature;

LTPRI2 LIT: linear temperature-programmed retention index for compounds from column 2 from literature.

The positive identification of selected compounds was highlighted in bold font.

**Table 5:** Compounds detected in raw spirit samples.

Name	RT/LTPRI1	RT/LTPRI2	LTPRI1 LIT	LTPRI2 LIT	Descriptor
(Z)-3-Hexen-1-ol	42.63/859	46.74/966	852	960	Mossy, green, and fresh
(Z)-4-Heptenal	46.13/899	48.66/991	900	988	Creamy, sweet, and boiled potato
1,2-Benzenediol	77.77/1318	78.44/1440	1324	1455	—
**1-Hexanol**	44.12/876	48.66/991	870	980	Dry, floral, grossy, green, woody, and fruity
1-Hexen-3-ol	34.53/770	38.32/860	775	850	Green
1-Hydroxy-2-propanone	25.24/655	35.08/820	643	810	Caramelized and sweet
1-Octen-3-one	52.11/970	54.49/1069	979	1066	Herbaceous
2,3-Pentanedione	28.79/702	33.61/802	698	788	Creamy, fresh, fruity, and sweet
**2-Methyl-1-butanol**	31.92/737	37.55/857	739	852	Fruity and malty
**2-Methyl-1-propanol**	23.26/629	28.83/741	628	735	Alcoholic, bitter, and winey
**2-Methylfuran**	21.06/601	22.64/652	602	639	Burnt, solvent, metallic, and musty
2-Methylphenol	58.18/1045	69.44/1290	1054	1283	Phenolic
**2-Methylthiophene**	35.16/774	35.92/831	775	827	Sulphurous
2-Undecanal	80.83/1365	82.07/1504	1368	1503	Fruity, *geranium*, green and metallic
**Alpha-pinene**	49.46/939	45.49/941	937	945	Green, pine, camphor, and sweet
**Benzaldehyde**	50.53/951	54.90/1075	959	1086	Almond, woody, and burnt sugar
Beta-ionone	88.68/1487	88.30/1615	1490	1627	Floral, woody, and raspberry
Butanoic acid	39.77/827	46.74/966	816	970	Butter, rancid, and sweaty
Carvone	73.57/1255	74.52/1373	1253	1386	Minty and peppermint
**Decanal**	70.67/1212	69.44/1290	1206	1293	Aldehydic, burnt, floral, and green
Decanoic acid	78.58/1330	80.10/1469	1323	1476	Fatty, rancid, and soapy
Diethyl ether	16.77/479	16.98/526	491	532	—
**Ethanol**	14.85/424	18.47/572	437	564	Alcoholic
**Ethyl acetate**	21.56/607	24.14/676	609	673	Acidic, etheral, fruity, and orange
Ethyl hexanoate	54.39/997	52.80/1046	995	1061	Anise, apple, fruity, and sweet
Ethyl octanoate	69.45/1195	68.26/1271	1196	1260	Anise, floral, fresh, and leafy
Ethyl phenylacetate	71.81/1229	73.79/1361	1243	1370	Anise, cinnamon, floral, and spicy
Furfural	40.93/840	46.94/966	827	972	Almond, bread, and sweet
Geosmin	85.79/1442	83.14/1523	1430	1513	Beet and earthy
Geranial	74.70/1272	76.83/1412	1270	1416	Citrus, floral, lemon, and minty
**Hexadecane**	96.54/1605	87.18/1595	1600	1600	Alkane, fusel, fruity, and sweet
**Hexanal**	36.62/791	41.31/897	795	883	Grassy, green, herbaceous, and leafy
Hexanoic acid	52.84/979	62.10/1177	990	1186	Fatty, rancid, and sweaty
Hexyl acetate	55.56/1012	56.21/1092	1011	1083	Citrus, fruity, green, and spicy
Hexyl butyrate	68.38/1180	67.11/1253	1191	1257	Apple, fruity, and green
Hexyl isobutyrate	65.11/1136	63.90/1203	1150	1208	Green
Indole	76.44/1297	85.12/1558	1295	1549	Burnt, earthy, floral, and jasmine
**Limonene**	56.84/1028	54.49/1069	1033	1061	Citrus, fruity, minty, and peely
**Linalool**	62.89/1106	62.96/1189	1099	1195	Floral, fruity, green, and lavender
Methyl butanoate	31.22/729	31.74/778	717	784	Ester, etheral, green, and sweet
Methyl-2-propenoate	22.20/616	24.82/687	611	680	—
n-Butanol	26.04/666	29.84/754	651	778	Fermented and fruity
**Nonane**	46.49/903	41.31/897	900	900	Alkane and fusel
P-Cresol	60.39/1073	71.49/1323	1072	1312	Phenolic and smoky
**Pentadecane**	90.40/1514	82.07/1504	1500	1500	Alkane, fusel, and mild green
Pentanol	34.68/766	39.87/873	767	879	Anise, fruity, and green
Propenal	15.61/446	18.18/563	450	566	—
Propyl acetate	29.52/710	31.36/773	708	775	Fruity, ketonic, sweet, and solvent
Terpinolene	61.46/1087	58.14/1120	1088	1112	Fruity, pine, herbaceous, and sweet
tert-Butylmethylether	19.08/545	19.79/607	546	600	—
**Tridecane**	77.16/1308	70.34/1304	1300	1300	Alkane, citrus, fruity, and fusel
**Undecane**	62.24/1097	57.03/1104	1100	1100	Alkane and fusel

RT1: retention time in column 1 (MXT-5);

LTPRI1: linear temperature-programmed retention index for compounds from column 1;

RT2: retention time in column 2 (MXT-1701);

LTPRI2: linear temperature-programmed retention index for compounds from column 2;

LTPRI1 LIT: linear temperature-programmed retention index for compounds from column 1 from literature;

LTPRI2 LIT: linear temperature-programmed retention index for compounds from column 2 from literature.

The positive identification of selected compounds was highlighted in bold font.
